# Smells in Sustainable Environments: The Scented Silk Road to Spending

**DOI:** 10.3389/fpsyg.2021.718279

**Published:** 2021-08-20

**Authors:** Jasper H. B. de Groot

**Affiliations:** Department of Social and Cultural Psychology, Behavioural Science Institute, Radboud University, Nijmegen, Netherlands

**Keywords:** sustainable, consumer behavior, odors, field study, expenses, cognition, emotion

## Abstract

Humanity's demand for ecological resources and services exceeds what earth can regenerate in that year, creating an urgent need for more sustainable behavior. Here, the focus is on a particular factor that so far has been overlooked in facilitating sustainable behavior, namely smell. The two-fold aim of this study was (i) to investigate whether ambient scent could enhance customers' subjective experience and spending behavior in a sustainable environment, and (ii) to elucidate the affective and cognitive pathways from scent to spending. To test this, a double-blind field experiment was designed where customers of a second-hand clothing store (*N* = 57) could face one of three conditions: fresh linen scent (pleasant and semantically priming “clean clothing” increasing the products' value), vanilla sandalwood scent (pleasant control odor), or regular store odor (odorless control). Buttressed by prior research, the fresh linen scent was expected to cause the strongest increase in spending behavior due to its positive semantic association with the product (i.e., clean clothing). The results indeed showed that fresh linen scent almost doubled consumer spending vs. the odorless control *and* the pleasant control odor. Other factors potentially affecting consumer behavior (e.g., weekday, weather, odor awareness) were uncorrelated. Whereas a conceptually-driven mediation analysis showed that only fresh linen scent increased mood and evaluations of the store, staff, and products, these variables did not mediate the relation between scent and spending. An explorative structural equation model suggested cognitive priming to be mainly responsible for increasing consumers' spending in the fresh linen condition by enhancing the general store evaluation. Further support is needed to corroborate the indirect findings that specific scents can follow a “cold” semantic road and a “hot” affective road to spending. At minimum, consumers are no “zombies” that empty their pockets in the presence of whatever odor; the smell needs to have a meaningful link to the (sustainable) context at hand to influence consumer behavior.

## Introduction

Earth Overshoot Day (EOD) refers to the date on which humans' demand for ecological resources and services has exceeded what planet earth can regenerate in that year (Earth Overshoot Day, 2021). Alarmingly, this date has been creeping up the calendar every year, from September 23 in 2000 to July 29 in 2019 (Earth Overshoot Day, 2021). As carbon emissions form the largest driver of today's overall ecological footprint and climate change, one can imagine the huge impact of global trade and the shipping of goods like clothing on the environment. One of the most polluting industries in the world is the fashion industry (Howell, 2021). Even before transportation, the production of new clothes involves 1.2 billion tons of greenhouse gasses (Beall, 2020). After “consumption,” disposal forms another huge problem. Globally, the 92 million tons of textile waste each year make for one garbage truck full of clothes being unloaded every second (Beall, 2020). Synthetic clothing is especially problematic taking 20–200 years to fully decompose, releasing harmful greenhouse gasses like methane in the process which fuels global warming further (McCarthy, 2018). To overcome these problems and prevent irreversible damage to the environment or loss of function in natural systems, sustainable consumption is needed.

How can sustainable consumption be facilitated? The need for long range shipping and the unsustainable production and disposal of clothing is reduced when more individuals recycle their clothing and buy second-hand clothing in local stores. More and more people seem to become aware of their individual contribution to a more sustainable world, met by a growing number of suppliers of local used products like second-hand clothing. Yet, what factors could persuade people to actually make more sustainable choices in the store?

A deliberate and predictable intervention of changing people's behavior by modifying the cues in the physical and/or social context in which they act is called *nudging* (Thaler and Sunstein, 2008; Marchiori et al., 2017). Nudges are believed to subtly influence consumer behavior by instigating non-conscious processes. As such, stimuli that easily escape awareness may be particularly potent, like smells. Although a number of field studies on scent marketing have demonstrated the potent effects of smells on consumer experience and behavior (e.g., Spangenberg et al., 1996; Fiore et al., 2000; Morrin and Ratneshwar, 2000; Chebat and Michon, 2003; Davies et al., 2003; Bosmans, 2006; Bradford and Desrochers, 2009; Doucé and Janssens, 2011; Morrison et al., 2011; Vinitzky and Mazursky, 2011; Doucé et al., 2014; for reviews, see e.g., Spence, 2015, 2020), *chemonudges* have remained neglected in the context of sustainable consumer behavior and their mechanisms are relatively unknown. This is surprising because through a process called “priming” (Smeets and Dijksterhuis, 2014), smells may activate semantic associations that could facilitate sustainable behavior (cf. Bimonte et al., 2020, for visual priming effects on pro-environmental behavior). If a scent prime proves to be effective in a second-hand clothing store by stimulating customers' sustainable shopping behavior, simple applications like scent diffusers can have a modest yet significant contribution in reducing the urgent environmental problems posed by unsustainable fashion.

### Literature Review: Smells in the Built Environment

The business environment is one of fierce competition between companies eager to gain customer loyalty and to maximize sales. In this competitive atmosphere, marketeers continually search for new ways to enhance customers' spending. One way to influence consumption is by creating a pleasant atmosphere by emitting a fragrance (Spence, 2015, 2020). For decades, bakeries, coffee houses, and restaurants have used their food scents to attract customers. Nowadays, an even greater variety of companies work with synthetic fragrances to inspire “consumption” (Emsenhuber, 2009). Marketeers have become increasingly aware of the role scent can play in differentiating brands and improving customers' sense of well-being in marketplace settings (Morrin, 2009), and the term *scent marketing* emerged: “using scents to set a mood, promote products, or position a brand” (Vlahos, 2007). Several large players in the commercial fashion market (like Primark, H&M, Scotch and Soda, and ZARA) have already applied scent marketing (AirAroma Scent Marketing, 2016; MoodMedia, 2016), but the effectiveness of scents in these settings cannot be assessed without scientific publications.

What are the elements that make for a potential effective *chemonudge*? Based on the broader literature reviewed below, smells' potency arguably lies in their capacity to affect our behavior subconsciously, aside from inducing deep-seated emotions and durable semantic associations—mechanisms that are facilitated by the distinct smell brain anatomy.

#### Smells and Awareness

Before a person consciously notices a scent, molecules have already attached to odorant receptors in the nose and reached among the phylogenetically oldest brain regions conserved in humans to produce an often immediate, instinctive reaction (Zaltman, 2003; Vlahos, 2007). Indeed, the human sense of smell provides the “minimal neuroanatomy for a conscious brain” (Morsella et al., 2010; in Keller, 2011). Smells can thus easily remain beneath the radar of conscious reporting, and its exactly at this stage when our behavior is most affected, as lab studies have shown. Our social preferences (face likeability ratings) are guided most strongly when we are presented with a subliminal positive or negative smell (Li et al., 2007). Li et al. (2007) argued that subliminally presented smells prevent strategic processes like “cognitive discounting” from occurring that could otherwise reduce the smell's influence. Another factor contributing to less conscious attention for smells are neurocognitive limitations making it hard for most (Westerners, at least) to put smells into words (Olofsson and Gottfried, 2015; cf. Majid and Kruspe, 2018). Because of this, smell effects can remain under the hood and be relatively non-distracting. Retailers can effectively take advantage of this by applying scents to enhance customer experience and stimulate purchases (Fiore et al., 2000) without distracting the consumers' attention from other stimuli, like the clothes they are looking at Davies et al. (2003).

#### Smells and Affect

Smells have an intimate link with feelings that is unique among the senses. There is close structural overlap between brain regions processing smell and those devoted to emotion processing (Gottfried, 2006). Odors also evoke the strongest emotional autobiographical memories (see Hackländer et al., 2019, for a review), and these (memory-related) affective reactions occur before cognitive processes take place. Ample research has shown that scents can alter our mood (Ehrlichman and Halpern, 1988; Baron, 1997; Lee et al., 2011). Other studies have shown that the mood of customers has a positive impact on their evaluation of the store and its staff (e.g., Dawson et al., 1990; Swinyard, 1993; Tice et al., 2001; Arnold et al., 2005; Morrison et al., 2011). Positive affect associated with a pleasant ambient scent also transfers to the items being evaluated (Morrin and Ratneshwar, 2000; Doucé and Janssens, 2011). However, some researchers have argued that for the positive affect to transfer, the scent must be congruent with the product category or at least match in arousing qualities (e.g., Mitchell et al., 1995; Spangenberg et al., 1996; cf. Bosmans, 2006, for a different view). According to *affect-as-information theory*, our subjective feelings can serve as a criterion for our decisions (Schwarz and Clore, 1983; Schwarz, 2011). For instance, if a smell induces a positive feeling, this feeling could transfer to an item within a store and inform a buy decision. However, for affect-as-information to work, individuals typically need to identify their affective state as potential criterion for decision making, while not discounting the influence of affect (Albarracín and Kumkale, 2003). Hence, humans are no zombies that buy every product in a store once a pleasant smell is released—reality is more complex, and cognition plays a role as well.

#### Smells and Cognition

Because odor processing does not end at the more primitive and emotional limbic system, another possibility is that ambient scents affect consumers through their connection with semantic knowledge (Degel et al., 2001). Lab studies have already shown that certain scents, like the smell of all-purpose cleaner, can activate cleaning-related concepts in our brain and instigate actual cleaning behavior, even without our awareness (Holland et al., 2005). The same phenomenon was replicated in a field setting, as de Lange et al. (2012) found that train passengers produced less litter in citrus-scented train wagons compared to non-scented wagons. In another field experiment, Doucé et al. (2014) found that a pleasant ambient scent had a negative effect on customers' product evaluation in a messy store; yet, this effect disappeared when the pleasant scent was associated with neatness. Learned cognitive associations with a scent are thus likely to influence consumer behavior aside from the more direct emotional effects.

To make matters more complex, it should be noted that peoples' semantic scent-associations have been formed and become reactivated in a particular multisensory context. A recent Virtual Reality (VR) study underlined that odors interact in complicated ways with the context in shaping human behavior (de Groot et al., 2020a). In particular, VR was applied to create a realistic, immersive, yet controlled multisensory context (laundry scenario), which was contrasted with a sterile, non- immersive, traditional lab setting. Using a computer-controlled odor delivery device (olfactometer), participants were exposed to three odors: (i) one odor semantically related to cleaning (detergent smell); (ii) one equally pleasant cleaning-unrelated odor (vanillin); and, (iii) no odor (regular room air). Although positive affect may also fuel a person's motivation (Custers and Aarts, 2005), the results showed that the pleasant odor vanillin could not enhance a persons' motivation to clean (de Groot et al., 2020a). Notably, the multisensory context (a VR laundry scenario) and smell (laundry odor) interacted and both were required to fuel motivated and effective cleaning behavior, objectively and subjectively (de Groot et al., 2020a).

### The Present Research

The aim of the current research was to examine whether smells can facilitate sustainable behavior. Given the environmental problems associated with the fashion industry, the setting was chosen to be a second-hand clothing store in a large city in the Netherlands. The results of a pre-test (*N* = 79) revealed that hygiene was the second highest rated factor in a person's decision to buy second-hand clothing (53.2%), after lack of damage (70%), but before style (49.4%) and price (32.9%). Hence, to test whether consumer spending in a second-hand clothing store was related to the mere pleasantness of a diffused odor (affect) or through its positive semantic associations with clean clothing items (cognition), customers could smell a fresh linen scent, an equally pleasant vanilla sandalwood odor, or regular store odor. Based on prior research (e.g., Chebat and Michon, 2003; Holland et al., 2005; de Groot et al., 2020a), the *main* hypothesis was that fresh linen scent would increase spending in a second-hand clothing store compared to vanilla sandalwood and regular store odor, due to a combination of affective and cognitive factors.

**Figure 1:**
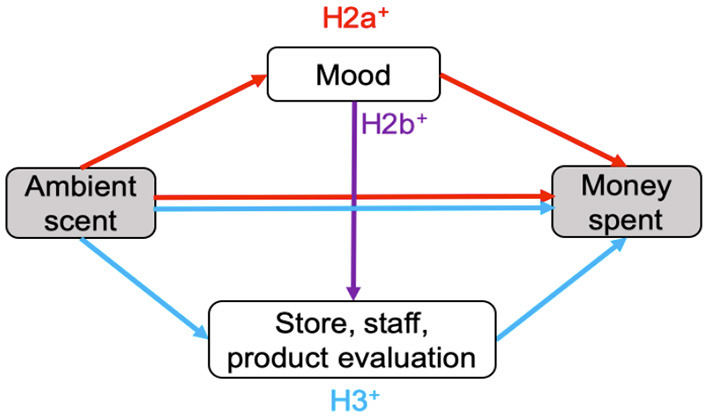
Conceptual model with hypothetical pathways from ambient scent to spending. “Hot” affective route in red and “cold” semantic route in blue.

Second, although various studies have shown effects of ambient scent on consumer behavior (e.g., Chebat and Michon, 2003; Morrison et al., 2011; Vinitzky and Mazursky, 2011), the precise mechanisms have generally remained elusive (Spence, 2020). Here, I propose a conceptual model (Figure 1) rooted in prior research to clarify the pathways from scent to spending in the context of sustainable behavior (hypothesis 2a, 2b, 3). First, an ambient scent like fresh linen (pre-tested to be pleasant and associated with cleanliness, see Materials and Methods) is expected to induce a positive mood (e.g., Ehrlichman and Halpern, 1988; Baron, 1997; Khan et al., 2007; Lee et al., 2011; Haehner et al., 2017; Spence, 2020) vs. the regular store odor. Using their feelings as information (Schwarz and Clore, 1983), this positive mood is believed to mediate the amount of money spent on clothing items (H2a) and to transfer to a better evaluation of the store, staff, and products (H2b) (store and staff: Dawson et al., 1990; Swinyard, 1993; Tice et al., 2001; Arnold et al., 2005; Morrison et al., 2011; products: Morrin and Ratneshwar, 2000; Doucé and Janssens, 2011). Aside from “hot” affective processes, the fresh linen scent is expected to positively affect store, staff, and product evaluation through “cold” cognitive processes (Degel et al., 2001; Doucé et al., 2014), which are expected to mediate the relation between scent and spending (H3).

## Materials and Methods

### Participants and Design

A total of 57 customers between 18 and 65 years (*M*_age_ = 42.32 years, *SD* = 14.05) participated voluntarily in this research. Of the 57 participants, 53 identified as “female,” and 4 as “male.” This gender imbalance fits with research showing that females are generally more interested in second-hand clothing stores and vintage stores than males (Cervellon et al., 2012). There were no formal inclusion criteria, and there was no incentive. Participants were asked to fill out a questionnaire directly after having bought an item at the “Green Label Store” (response rate: 16.8%). The Green Label Store is a second-hand store in the city center of Utrecht, the 4th largest city in the Netherlands (357,719 inhabitants), which mainly sells second-hand clothes.

Participants enrolled in a double-blind between-subjects design, with Scent Condition being the sole experimental factor (three levels: fresh linen, vanilla sandalwood, no odor). No evidence was found that age differed significantly across conditions (fresh linen, *n* = 21; *M* = 47.43 years, *SD* = 11.85; vanilla sandalwood: *n* = 19, *M* = 41.47, *SD* = 16.06; no odor: *n* = 17, *M* = 36.94, *SD* = 12.61), *F*_(2, 54)_ = 2.85, *p* = 0.067.

### Materials and Measures

#### Odors

The independent variable in this research was scent. To create the scent conditions, the fresh linen scent and vanilla sandalwood odor were pre-tested for their suitability. During the “no odor” condition, the regular store odor was maintained because no odor was diffused.

##### Fresh Linen

A pretest was conducted to examine if fresh linen scent would “prime” customers with the idea that products in the second-hand store are hygienic. Participants in this pretest (*N* = 22) were asked to rate odor pleasantness and its association with cleanliness on a 10-point scale. The results revealed fresh linen to be significantly above the midpoint of the scale with regard to pleasantness (*M* = 7.55, *SD* = 1.33), *t*_(21)_ = 8.99, *p* < 0.001, as well as being associated with cleanliness (*M* = 8.27, *SD* = 0.99), *t*_(21)_ = 15.49, *p* < 0.001. Hence, fresh linen scent could influence customers' spending in our field experiment through an affective and/or cognitive semantic priming route. The molecules that made up “Fresh linen” scent are: alpha-Hexylcinnamaldehyde, 4-tert-Butylcyclohexyl acetate, 1-(1,2,3,4,5,6,7,8-Octahydro- 2,3,8,8-tetramethyl-2-naphthalenyl) ethanone, Hexyl salicylate, d-Limonene, 3 and 4-(4- Hydroxy-4-methylpentyl)-3-cyclohexene-1-carboxaldehyde, alpha-Methyl-1,3-benzodioxole-5- propionaldehyde (MoodMedia NL, Almere, the Netherlands).

##### Vanilla Sandalwood

Prior research indicated that vanilla is perceived as significantly non-fresh (Fenko et al., 2009), and both vanilla (Spangenberg et al., 2006) and sandalwood (Lwin and Morrin, 2012) are perceived a pleasant ambient smell in applied settings. Furthermore, the company ScentAir recommended vanilla sandalwood as a pleasant control scent based on their in-house data (MoodMedia NL, Almere, the Netherlands). The components of “Vanilla sandalwood” were: Vanillin; 1-(1,2,3,4,5,6,7,8-Octahydro-2,3,8,8-tetramethyl-2- naphthalenyl)ethenone; 3-(5,5,6-Trimethylbicyclo[2.2.1]hept-2-yl)cyclohexan-1-ol; 1,3,4,6,7,8-Hexahydro-4,6,6,7,8,8- hexamethylcyclopenta-gamma-2- benzopyran; p-t-Butyl-alpha-methylhydrocinnamic aldehyde; alpha-iso-Methylionone. No lab-based pilot test was carried out for this odor, which can be considered a limitation. *Post-hoc*, there were no differences in odor pleasantness between fresh linen and vanilla sandalwood in the field setting (see Results).

##### Odor Dispersion

**Figure 2:**
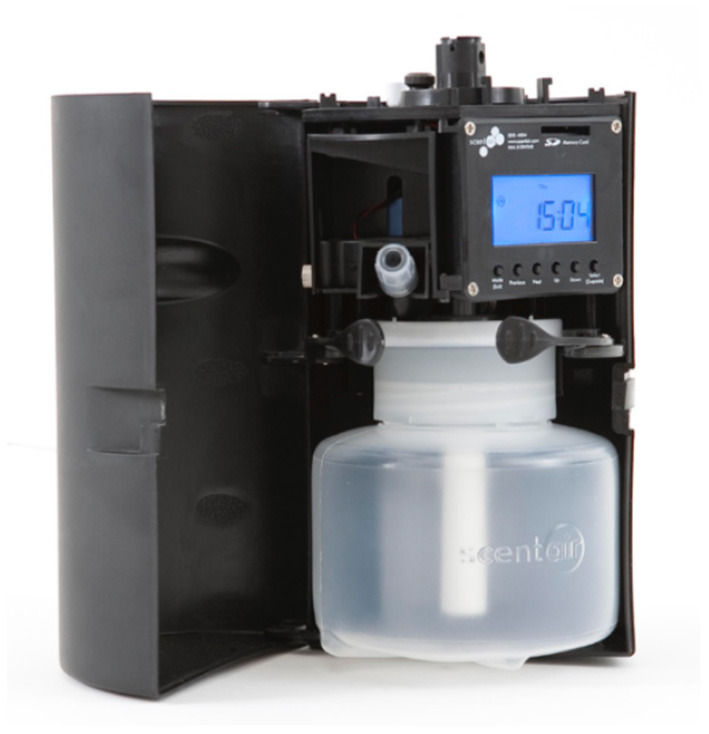
ScentDirect odor diffusion system.

The company ScentAir, part of MoodMedia (MoodMedia NL, Almere, the Netherlands) provided the scents and scent diffusion apparatus (Figure 2). Both companies have years of experience in real world scent diffusion. The patented scent diffusion system “ScentDirect SDD-4004” (height: 235 mm, diameter: 45 mm) is capable of diffusing scent in an area of 850 m^3^, which greatly exceeds the dimensions of the Green Label Store. It features advanced diffusion technology that converts liquid fragrance oil from cartridges (0825 Fresh Linen ON or 1807 Sandalwood Vanilla) into a fine, dry, invisible mist and releases it directly and consistently into the environment. To maximize the efficiency of scent delivery, the airflow in the store was determined by ScentAir. To cover the entire store with scent, ScentAir decided to install the ScentDirect diffuser about half a meter from the entrance. Customers were exposed to the smell during their purchase and during the completion of the questionnaire which was at the counter (located next to the entrance).

##### Scent Calibration: Awareness and Pleasantness

When a scent becomes too intense, people may become aware that their responses are being influenced by the scent and change their behavior (Bosmans, 2006). Based on their in-house research, ScentAir selected a scent intensity ensuring the scents were not too salient, quantified by ~25% of the customers noticing the scent and 75% not being aware of its presence. To verify this, a manipulation check was included at the end of the questionnaire. Participants were asked if they smelled a scent which was different from what they expected in the store. If they answered this question with “yes” they were asked to fill in what they thought they smelled. Only three individuals in the fresh linen condition guessed “laundry odor” or “fresh smell,” and only three individuals in de vanilla sandalwood condition guessed “sandalwood” or “sweet odor” (~15% of the sample). In both scent conditions, 27 individuals “*noticed a smell”* and provided smell ratings (of which 19 individuals mentioned the smell was *different from what they expected*). All 27 customers subsequently rated the scent they noticed on 7-point Likert scales. Importantly, the pleasantness ratings of fresh linen scent (*M* = 5.43, *SD* = 1.55; *n* = 14) was again significantly above the midpoint (3.5) of the scale, *t*_(13)_ = 4.66, *p* < 0.001, and virtually identical to vanilla sandalwood odor (*M* = 5.46; *SD* = 1.56; *n* = 13), *t*_(25)_ = −0.05, *p* = 0.957.

#### Questionnaire

Aside from asking the participant to report the amount of money they spent in the store, this 13-item questionnaire intended to assess on a 7-point Likert scale the customer's evaluation of the store environment (“Store evaluation”; four items), their evaluation of the products (“Product evaluation”; four items), their evaluation of the staff (“Staff evaluation”; two items), and the mood of the customer (“Mood”; three items). Of these 13 items, five items were selected based on their high factor loadings (>0.80) in a similar study by Chebat and Michon (2003), with the original source cited for each selected item. For “Store evaluation,” two items were selected based on prior research (“How boring/stimulating do you find this store”; “How (un)interesting do you find this store?”) (Fisher, 1974; cf. Chebat and Michon, 2003). Note, the question about liveliness was omitted; instead, it was asked: “What is your general impression of this store?,” and “What is your impression of the messiness of this store?.” For “Product evaluation,” one item was selected based on prior research (“What do you think of the quality of the products in the store?”) (Bellizzi et al., 1983; cf. Chebat and Michon, 2003). This item was complemented by three items that were more specific to the current context: “How hygienic are the products in this store?,” “How much trust do you have in the products being sold in this store?,” and “Did you doubt about your purchase?” (Product evaluation). Regarding “Mood,” two items were selected from prior research (“How (un)happy do you feel at this moment?”; “How annoyed/pleased do you feel at this moment?”) (Mehrabian and Russel, 1974; cf. Chebat and Michon, 2003). The items about satisfaction and feeling melancholic/contented were considered as superfluous for the current setting. In an attempt to capture the whole emotion experience (quality and quantity) in one scale, an extra item on “Mood” was added to target the arousal dimension going from relaxed to tense. In retrospect, this item would have better fitted a separate category of arousal (vs. valence) (see Results; Mehrabian and Russel, 1974; Chebat and Michon, 2003). Fourth, “Staff evaluation” was assessed using two items asking about staff friendliness and helpfulness (cf. Simmers and Keith, 2015).

After this 13-item questionnaire, control questions were asked to check if participants were aware that a different scent was diffused in the store and, if so, how they rated this scent on appropriateness, intensity, familiarity, and pleasantness. The final questionnaire can be found in the Supplementary Material (Appendix 1: Dutch questionnaire with key terms translated in English; and Appendix 2: the original untranslated questionnaire).

##### Reliability Analysis

A reliability analysis was done to verify the internal consistency of the originally constructed subscales (“Store evaluation,” “Staff evaluation,” “Mood,” and “Product evaluation”). From the initial 13 items, three had to be deleted from the various subscales to reach acceptable internal consistency levels (Table 1) (Gravetter and Wallnau, 2007).

**Table 1:** Cronbach's α before and after removing items from Store, Staff, Mood, and Product.

**Factor**	**Number of items**	**Cronbach's α**	**Cronbach's α if item deleted**	**Remaining number of items**
Store	4	0.458	0.867	3
Staff	2	0.735	0.735	2
Mood	3	0.051	0.730	2
Products	4	0.405	0.692	3

“Store” initially consisted of four items (α = 0.458); yet, as the store messiness item (#10) did not correlate well with the other store evaluation items (#1–3: *r*s > 0.035 < 0.154), this item was deleted (α = 0.867). “Staff” consisted of two items (α = 0.735); these items correlated well (*r* = 0.585) and could be retained. “Mood” initially consisted of three items (α = 0.051); yet, the experienced tension item (#5) correlated negatively with the other mood items (#7: *r* = −0.154; #13: *r* = −0.134). After deleting this item, internal consistency was acceptable (α = 0.781). Finally, the internal consistency of “Product evaluation” (four items: α = 0.405) was raised (α = 0.692) after deleting the purchase doubt item (#9), which did not correlate well with the rest (#4, #6, #11: *r*s > −0.074 < 0.114).

### Procedure

The experiment was conducted in a second-hand clothing store for seven consecutive weeks. The following scent condition order was maintained: “No odor” condition (week 1), “Fresh linen” scent condition (week 2–3), and “Vanilla sandalwood” scent condition (week 5–7). A person from the company ScentAir arranged proper scent diffusion. A gap week (week 4) was aimed to neutralize the smell to avoid odor mixtures. The experiment was double-blind: experimenters (aware of the study's hypothesis) were not present. Instead, store personnel received a hand-out with general information about the study design. While the staff knew that the regular store odor was altered in the two scent conditions, they were neither aware of the exact content, nor of the study's hypotheses. Employees were instructed to behave as naturally as possible during the experiment, to keep track of how many people entered the store and bought an item, and to ask a customer to voluntarily fill out a questionnaire after a purchase. At the end of each day, an experimenter collected the data.

### Statistical Analyses

Data and analyses are available here: https://osf.io/ax7yp/. The analyzed sample (*N* = 57: fresh linen: *n* = 21; vanilla sandalwood: *n* = 19; regular store odor: *n* = 17) consisted of individuals aged 18–65. By age 65, about half the population has noticeably impaired smell abilities (e.g., Kern et al., 2004; Wolfe et al., 2006). Three individuals self-reported a decreased sense of smell, and analyses were performed with and without these individuals to check for its impact.

Regarding the main effect of ambient scent on the mean total amount of money spent in a second-hand clothing store (hypothesis 1), it was first checked whether data were normally distributed. If so, a one-way ANOVA was conducted with scent condition as the sole factor. Following up on a main effect of scent condition, planned contrasts would verify the nature of the difference.

Regarding the relations from ambient scent to consumer spending (hypothesis 2a, 2b, 3), three questionnaire items had to be rescored because their scales were reversed: Item 5 “How do you feel at the moment?” Relaxed-Tense; Item 9: “Did you doubt about your purchase?”; Item 10: “What impression does the store make on you?” Not messy-messy. To maintain power, missing responses on the questionnaire (4.1%) were imputed in IBM SPSS 27, per scent condition, using regression imputation including a random error term. Then, a reliability analysis was done on all 13 items to check whether the subscales (store, product, staff, mood) were internally consistent (see Table 1, Results). To assess relations from the conceptual model (Figure 1), mediation analysis was performed with the PROCESS procedure for SPSS (Version 3.5.3) following the method from Hayes (2018). Within the PROCESS macro, model 4 was selected to test hypothesis 2a, 2b, and 3 with simple mediation. The significance of the indirect effect was tested using bootstrapping procedures. Specifically, unstandardized indirect effects were computed for each of 5,000 bootstrapped samples, and the 95% confidence interval (CI) was computed by determining the indirect effects at the 2.5th and 97.5th percentiles. Mediation was present if the 95% CI did not overlap with 0. Second, to explore a possible best fitting model between latent and observed variables in a data-driven way, structural equation modeling was performed in JASP (JASP Team, 2020).

## Results

### Scent and Amount of Money Spent

**Figure 3:**
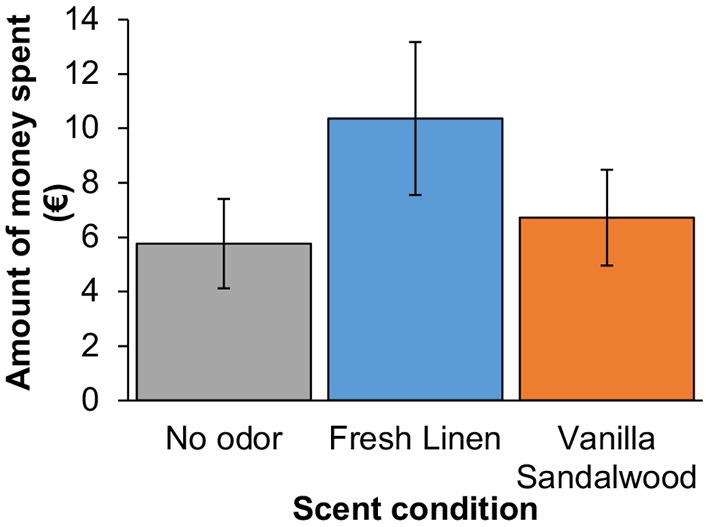
Mean total amount of money spent by customers in second-hand store as a factor of scent.

The first hypothesis entailed that exposure to a fresh linen scent would cause people to spend more money in a second-hand clothing store compared to no odor (regular store odor) and control odor exposure (vanilla sandalwood). As the data were normally distributed according to a Shapiro-Wilk test (*p*s > 0.126), a one-way ANOVA was conducted with condition (three levels: fresh linen, control, vanilla sandalwood) as between-subjects factor. Because Levene's test indicated significant non-homogeneity of variances, correction of the degrees of freedom was performed using Brown-Forsythe. The ANOVA showed a significant effect of condition, *F*_(2, 44.40)_ = 5.23, *p* = 0.009, η^2^ = 0.15. Planned contrast tests with degrees of freedom corrected (not assuming equal variances) indicated that, on average, customers spent significantly more money in total in the fresh linen scent condition (*M* = €10.36, *SD* = €6.44) vs. the no odor condition (*M* = €5.76, *SD* = €3.39), *t*_(31.42)_ = 2.83, *p* = 0.008, *d* = 0.87 [0.19–1.53], and vs. the vanilla sandalwood condition (*M* = €6.72, *SD* = €3.84), *t*_(33.1)_ = 2.20, *p* = 0.035, *d* = 0.68 [0.04–1.31] (Figure 3). No significant differences were found between the two control conditions (no odor vs. vanilla sandalwood): *t*_(34.00)_ = 0.79, *p* = 0.432.

Participants did not only spend more money in the fresh linen condition, but a descriptive analysis (there were not enough data points—days tested—for a formal analysis) also indicated that, with an average amount of 60 customers per day across conditions, the fresh linen scent witnessed a higher ratio of customers that bought an item (23.77 vs. 16%).

#### Controlling for Potential Confounds

Considering only the scent conditions (fresh linen, vanilla sandalwood), 19 individuals reported to have smelled a different odor than expected in this store, whereas 21 did not. An independent samples *t*-test revealed that reported awareness of a different-than-expected odor did not significantly impact spending behavior, *t*_(38)_ = 0.922, *p* = 0.362. Three individuals in the fresh linen and three more in the vanilla sandalwood condition correctly guessed the odor. Repeating the ANOVA without these individuals still gave a significant effect of scent condition, despite the smaller sample, *F*_(2, 48)_ = 3.41, *p* = 0.041, η^2^ = 0.12.

Murray et al. (2010) found that customer spending was related to more exposure to sunlight and concomitant decreased negative affect. In the present research, the higher amount of money spent in the fresh linen condition could not be due to extraneous factors like the average outside temperature, hours of rain, or hours of sunshine. A Kruskal-Wallis test indicated significant differences across conditions on all variables [average outside temperature: *H*(2) = 36.03, *p* < 0.001; hours of rain: *H*(2) = 32.16, *p* < 0.001; hours of sunshine: H(2) = 18.13, *p* < 0.001]. A Mann-Whitney test indicated that the average outside temperature was lower for the fresh linen condition (*Mdn* = 10.1°C) than for the vanilla sandalwood condition (*Mdn* = 17.6°C), *U* = 16, *p* < 0.001. The no odor control condition yielded the lowest average outside temperature (*Mdn* = 8.9°C) (vs. fresh linen: *U* = 99, *p* = 0.018; vs. vanilla sandalwood: *U* = 6, *p* < 0.001); the highest rain hours (*Mdn* = 2.5) vs. fresh linen (*Mdn* = 0), *U* = 99, *p* = 0.018, and vs. vanilla sandalwood (*Mdn* = 0), *U* = 23, *p* < 0.001; and the lowest sunshine hours (*Mdn* = 2.4) compared to fresh linen (*Mdn* = 8), *U* = 31, *p* < 0.001, and compared to vanillin sandalwood (*Mdn* = 13), *U* = 68, *p* = 0.003. All other comparisons were non-significant. Finally, there were no significant correlations between amount of money spent and average outside temperature, *r*(55) = −0.06, *p* = 0.682, hours of sunshine, *r*(55) = 0.16, *p* = 0.242, and hours of rain, *r*(55) = −0.13, *p* = 0.348.

Data were collected from Tuesday until Saturday and because day of the week could influence spending behavior (e.g., Stewart et al., 2012), weekday was also controlled for (cf. Murray et al., 2010). The analysis revealed that day of the week could not significantly predict spending behavior (*F* < 1; Tuesday: *M* = €8.45, *SD* = €6.29; Wednesday: *M* = €8.37, *SD* = €4.68; Thursday: *M* = €8.64, *SD* = €6.21; Friday: *M* = €8.78, *SD* = €3.36; Saturday: *M* = €5.09; *SD* = €3.74); adding weekday as a covariate to the main analysis did not eradicate the effect of scent on spending, *F*_(2, 53)_ = 4.02, *p* = 0.024, η^2^ = 0.12. The time customers spent in the store may also impact purchasing behavior (e.g., Freathy and O'Connell, 2012); yet, as this factor was not recorded because tracking each customer appeared too difficult in the current set up, it could not be controlled for. Hence, it is possible that customers in the fresh linen condition spent longer in the shop and therefore spent more money, and whether this is driven by the odor is unknown.

#### Short Summary

Controlling for a number of confounds, customers spent significantly more money in the fresh linen scent condition compared to the vanilla sandalwood and no odor condition, which supports hypothesis 1. For hypothesis 2, a deeper dive into the mechanisms from “ambient scent” (vs. no scent) to spending behavior is required. It should be noted that because a few factors (e.g., customer dwell time) could not be accounted for, and because the sample size was rather small (fresh linen: *n* = 21; vanilla sandalwood: *n* = 19; regular store odor: *n* = 17), the indirect effects reported below should be interpreted with relative caution.

### From Scent to Spending: Exploring Hot and Cold Routes Through Mediation Analysis

**Figure 4:**
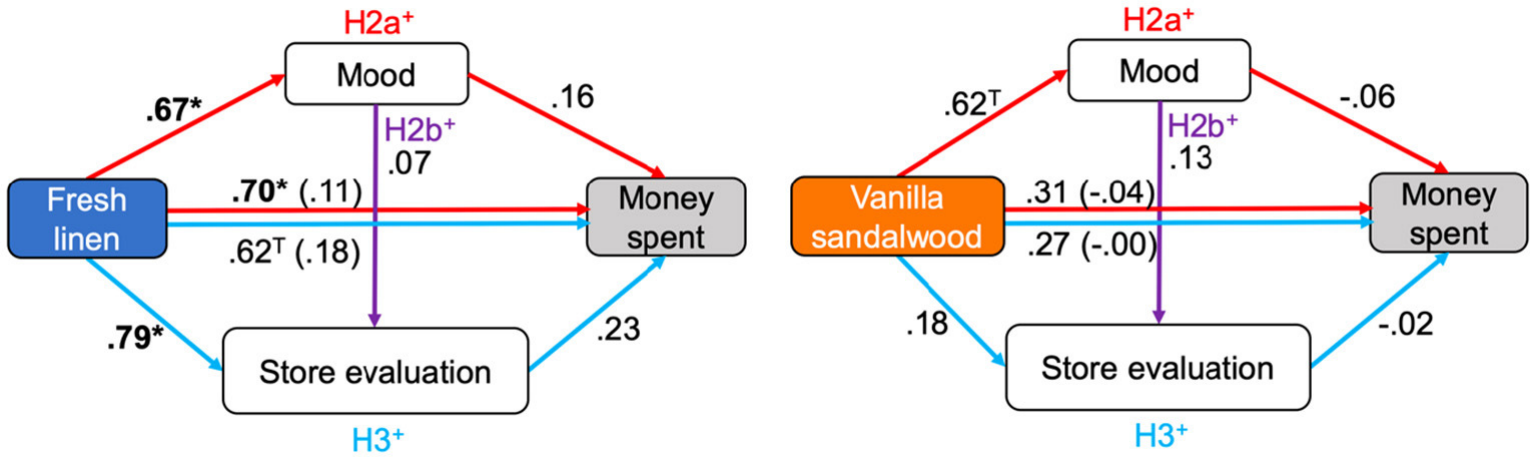
Conceptual model with standardized regression weights and significance indicated. Comparisons include fresh linen (left) and vanilla sandalwood (right) vs. regular store odor. *P < 0.05.

To test the pathways from scent to spending in the context of sustainable behavior based on the prior literature (hypothesis 2a, 2b, 3), simple mediation analyses were conducted (Figure 4).

First, ambient scent (vs. regular store odor) was expected to increase spending through elevating the customer's mood (H2a). Indeed, fresh linen scent significantly increased mood (*b* = 0.74, *t*_(36)_ = 2.15, *p* = 0.038, 95% CI: 0.04, 1.44]), whereas only a trend was found for vanilla sandalwood (*b* = 0.62, *t*_(34)_ = 1.94, *p* = 0.061, 95% CI [−0.03, 1.38]). Whereas the direct path from fresh linen to money spent was significant (*b* = 3.99, *t*_(36)_ = 2.17, *p* = 0.037 [95% CI: 0.26, 7.73]), the indirect path did not reach significance (*b* = 0.61 [95% CI: −0.57, 2.01]). Only 13.19% of the total effect of fresh linen on money spent was explained via (explicit) mood. In contrast, there was no direct effect of vanilla sandalwood on money spent (*b* = 1.10, *t*_(34)_ = 0.85, *p* = 0.401 [95% CI: −1.53, 3.73]; indirect effect: *b* = −0.15 [95% CI: −1.19, 0.49]. In sum, whereas fresh linen scent increased mood and caused a higher amount of money to be spent, mood did not significantly mediate the relation between scent and spending.

Second, positive mood was expected to mediate the relation between scent and evaluations of the store, staff, and products (H2b). Because store evaluation correlated strongly with staff evaluation *r*(56) = 0.58, *p* < 0.001, and with product evaluation, *r*(56) = 0.55, *p* < 0.001, [staff-product: *r*(56) = 0.41, *p* = 0.002]; to prevent that several analyses had to be carried out, mediation analyses were carried using the most strongly correlated and most encompassing variable store evaluation. Aside from the path from scent to mood (presented above) being significant (fresh linen) or just not-significant (vanilla sandalwood), the pathway from mood to store evaluation was neither significant for fresh linen (*b* = 0.04; *t*_(36)_ = 0.43, *p* = 0.670 [95% CI: −0.16, 0.24]), nor for vanilla sandalwood (*b* = 0.13; *t*_(34)_ = 1.26, *p* = 0.218 [95% CI: −0.08, 0.34]). In the absence of significant direct and indirect effects in this model, the conclusion is that there is no evidence that mood mediated the relation between scent and store, staff, and product evaluations.

Third, through semantic priming, the fresh linen scent (but not vanilla sandalwood) was expected to enhance store, staff, and product evaluations, which would mediate the relation between the scent and spending (H3). The path from scent to store evaluations indeed was only significant for fresh linen (*b* = 0.52; *t*_(36)_ = 2.60, *p* = 0.013 [95% CI: 0.11, 0.93]), and not for vanilla sandalwood (*b* = 0.11, *t*_(34)_ = 0.54, *p* = 0.592 [95% CI: −0.31, 0.54]). The paths from store evaluation to spending were not significant (fresh linen: *b* = 1.99; *t*_(36)_ = 1.41, *p* = 0.166 [95% CI: −0.87, 4.85]; vanilla sandalwood: *b* = −0.14, *t*_(34)_ = −0.14, *p* = 0.890 [95% CI: −2.20, 1.92]). For vanilla sandalwood, neither the direct effect (*b* = 0.97, *t*_(34)_ = 0.79, *p* = 0.437 [95% CI: −1.54, 3.49]), nor the indirect effect were significant (*b* = −0.02 [95% CI: −0.73, 0.31]). Despite a significant *total* effect from scent to spending for fresh linen (*t*_(36)_ = 0.012 [95% CI: 1.09, 8.11]), the direct effect became non-significant (*b* = 3.55, *t*_(36)_ = 1.91, *p* = 0.064 [95% CI: −0.22, 7.33]), and the confidence intervals of the indirect effect overlapped with 0, rendering it non-significant (*b* = 1.04 [95% CI: −0.28, 3.25]). Still, in terms of effect size, the indirect pathway explained 22.71% of the significant total effect. In sum, fresh linen scent (vs. control odor, odorless control) did affect store evaluations; yet, these explicit evaluations insufficiently mediated the effect from scent to spending.

**Figure 5:**
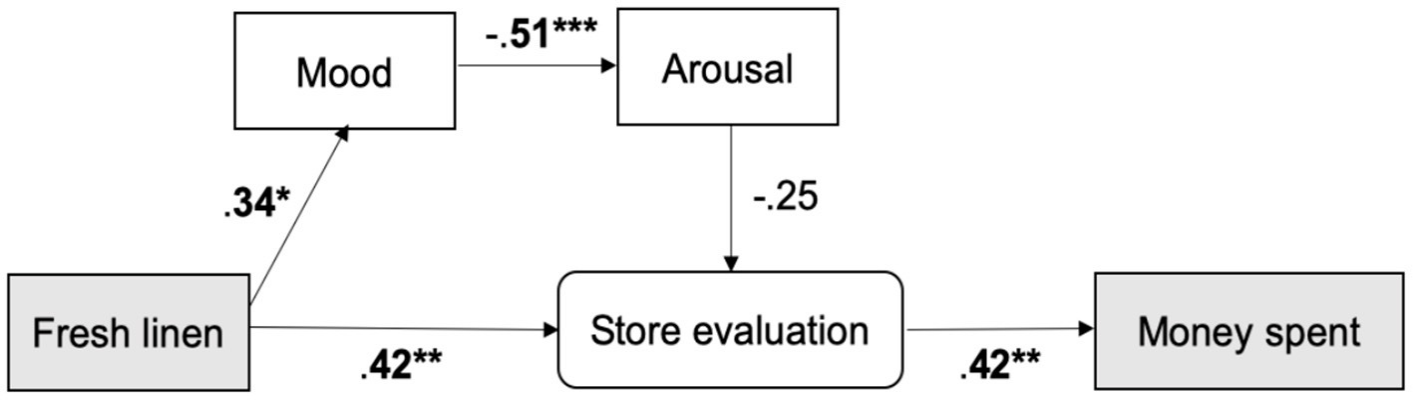
Best fitting model according to structural equation modeling. Store evaluation is a latent variable in this model measured with three items.

For exploratory purposes, structural equation modeling was performed to select the best fitting model from fresh linen scent to spending behavior in a data-driven way (Bollen and Long, 1993). Note that in this model, arousal appears a factor. No a priori expectations were set regarding arousal, but in this data-driven model the possibility for its inclusion was left open. Also, store evaluation, product evaluation, and staff evaluation were decomposed relative to the mediation analysis to see what the model would select as the best fitting predictor of money spent on second-hand clothing. The best fitting model [Goodness of fit Index (GFI) = 0.924; Comparative Fit Index (CFI) = 1.00; Root Mean Square Error of Approximation (RMSEA) = 0.00] (Hu and Bentler, 1999) contained a significant semantic route from fresh linen scent to “store evaluation” (*z* = 2.73, *p* = 0.006) and from “store evaluation” to customers' spending (*z* = 2.65, *p* = 0.008) (Figure 5). The results differ from the mediation analysis (Figure 4), because no direct pathway from scent to money spent was modeled (cf. Chebat and Michon, 2003), which would otherwise reduce explanatory variance. SEM also showed a significant route from fresh linen scent to mood (*z* = 2.26, *p* = 0.024), and inversely from mood to arousal (*z* = −3.69, *p* < 0.001), reflected by the experienced tension item. The path from arousal to “store evaluation” was not significant (*z* = −1.67, *p* = 0.095). Hence, there was neither a significant direct nor indirect pathway from mood to spending. According to SEM, a fresh linen scent (vs. control) resulted in higher store evaluations and mood, but only higher store evaluations led to more spending.

## Discussion

The main aim of this study was to investigate whether the diffusion of ambient scent would increase sustainable behavior in the form of customers spending more money in a second-hand clothing store. The secondary aim was to elucidate the mysterious ways in which ambient scent increases actual spending behavior in this environment, through “hot” affective processes, “cold” cognitive processes, or both. Although various studies have shown effects of ambient scent on consumer behavior (e.g., Chebat and Michon, 2003; Morrison et al., 2011; Vinitzky and Mazursky, 2011), the exact mechanisms from scents to spending have generally remained elusive (Spence, 2020). A field experiment was set up to test this. Based on prior research, a conceptual model (Figure 1) was devised to test the pathways from scent to spending in the context of sustainable behavior in a theory-driven way using mediation analysis. Aside from that, a structural equation model was created to explore which model would best fit the data in an unbiased, data-driven way. Mediation analysis showed that only a fresh linen scent (vs. vanilla sandalwood and regular store odor) could increase mood and evaluations of the store; yet, these variables did not mediate the significant link between fresh linen odor and spending. The data-driven model, however, suggested that fresh linen scent mainly influenced customers' behavior through a “cold” semantic route, a direction that warrants further scrutiny in a more powerful experiment using more sensitive, implicit measures of mood and cognition.

The *main* hypothesis was that, due to a combination of cognitive and affective factors, fresh linen scent would cause customers to spend more money in a second-hand clothing store compared to those smelling vanilla sandalwood (pleasant control odor) and the regular store odor (odorless control). Indeed, the results showed a large and medium-to-large effect of fresh linen scent on consumer spending behavior vs. the odorless control and pleasant control odor, respectively. Fresh linen scent almost doubled the amount of money that was spent on second-hand clothing. The reported effects were independent of odor awareness, odor pleasantness, and extraneous factors like day of the week and weather conditions.

The secondary hypotheses were aimed at the specific relations between ambient scent and spending behavior, including a person's mood, their evaluation of the store, staff, and products. Hypothesis 2a stated that compared to the regular store odor, an ambient scent like fresh linen (pre-tested to be pleasant and associated with cleanliness) would induce a positive mood (e.g., Ehrlichman and Halpern, 1988; Baron, 1997; Khan et al., 2007; Lee et al., 2011; Haehner et al., 2017; Spence, 2020), which would mediate the relation between scent and spending following affect-as-information theory (Schwarz and Clore, 1983). Whereas fresh linen scent indeed positively influenced mood (and only a trend was found for vanilla sandalwood, although both odors were not rated differently in pleasantness), there was no evidence that mood mediated the relation between scent and spending. Second, positive mood was believed to enhance evaluations of the store, staff, and products (H2b) (Dawson et al., 1990; Swinyard, 1993; Morrin and Ratneshwar, 2000; Tice et al., 2001; Arnold et al., 2005; Doucé and Janssens, 2011; Morrison et al., 2011). This hypothesis was not supported, as odor-induced mood could not predict higher evaluations of the store, staff, and products. Third, there was partial support for the hypothesis that only fresh linen scent would enhance store, staff, and product evaluations through semantic priming (H3) (Degel et al., 2001; Doucé et al., 2014): whereas fresh linen scent indeed enhanced store, staff, and product evaluations (and vanilla sandalwood did not), there were no significant mediation effects of ambient scent on spending via these evaluations. Explorative structural equation modeling also did not show a significant pathway from scent to spending via mood, whereas fresh linen scent did increase store evaluations, and higher store evaluations caused more money to be spent. These combined results suggest that fresh linen scent may have influenced consumers relatively more through a “cold” cognitive route that is based on semantic associations with the smell. These findings dovetail with Chebat and Michon (2003), who noted that “the cognitive effect of ambient scent [on consumers' spending] primarily passes through the perception of the retail environment”.

The results add to a growing number of studies highlighting the importance of cognition and context in shaping (multisensory) perception. Using VR, a recent study has shown that motivated goal-directed behavior as induced by odors is not related to odor pleasantness, but it only works for odors that have a semantic relation to the behavior, and this effect only occurs in a realistic, multisensory context (de Groot et al., 2020a). Prior to that study, others had shown that a cleaning-related odor (citrus) increased cleaning behavior, both in a lab environment (Holland et al., 2005) and in a field study (de Lange et al., 2012). At that point, it was unknown whether this form of semantic odor priming followed a reflex-like stimulus–response mechanism, with prototypical (cleaning) behavior always following (cleaning-related) odor exposure. However, such a perspective fails to account for the top-down mediating role that cognitions (“inspired” by context) have on the link between perception of scent and action. In the present research, this perception-action link was observed to operate via enhanced evaluations of the store, irrespective of mood. Although we cannot rule out effects of increased mood by ambient scents like fresh linen because the absence of evidence does not mean the evidence of absence, it does seem the present findings are best intelligible from situated cognition theory (cf. de Groot et al., 2020b). From this theory (e.g., Barsalou, 2016), scents are expected to fuel goal-directed behavior like buying clothing if the information a scent “communicates” (e.g., cleanliness) matches a person's criteria for buying second-hand clothing (e.g., needs to “feel” new, needs to be hygienic). What the current study thus has in common with more controlled lab studies is that it shows that human consumers are no “zombies” that immediately start spending money once they are exposed to ambient scent, but the smell has to convey a particular meaning in a particular context to be effective, operating through cognitive rather than affective processes, although a combination is not out of the question. This insight has practical implications, but the study also has a few shortcomings that are worthy of addressing.

### Limitations

One limitation is the potential constraint on generality posed by the characteristics of the current sample (Simons et al., 2017). The large majority of the sample consisted of females (93%) around the age of 40, who are regular visitors of a second-hand clothing store. Most (48.2%) are monthly or even weekly (28.6%) visitors of this or a similar store. Research has shown that females visit more stores and spend more time in stores then males (Dennis and McCall, 2005) and that second-hand clothing elicits more nostalgic feelings in females causing them to visit these stores more (Cervellon et al., 2012). Females also generally have a slightly better sense of smell than males (Sorokowski et al., 2019) and seem more susceptible to emotions associated with smells (e.g., de Groot et al., 2014). The current population may also differ from customers visiting more traditional commercial clothing stores, in the sense that the current sample may assign more meaning and value to second-hand clothing items (Cervellon et al., 2012). If so, this could have created a ceiling effect, reflected in the absent direct link between fresh linen scent and evaluations of the store, staff, and products; yet, fresh linen could still boost these evaluations in this sample. At present, we do not have evidence that our findings generalize to other populations like males. Yet, we have no reason to believe that fresh linen scent would *not* elicit spending behavior in males, because gender differences in smell abilities yield only small effect sizes (Sorokowski et al., 2019), and the spending behavior is expected to be triggered by representations of clean clothing items that are activated by fresh linen scent through mere association, and males are expected to have these associations as well (cf. Holland et al., 2005, showing all-purpose cleaner smell to induce cleaning behavior in a subsample of males also). However, it could be that individuals who wash more frequently will have a more positive association with clean clothing after smelling fresh linen scent. Although demographics like ethnic background were not collected, it is likely that the present research mainly consisted of Western, Educated, Industrialized, Rich, Democratic (WEIRD) individuals (Henrich et al., 2010). Although the scent priming mechanisms are assumed to be universal, its workings are dependent on the associations members of a certain culture have with a smell in order for it to effectively affect behavior.

Another limitation is in the mode of data collection, via a questionnaire. A questionnaire only taps into explicit process and it is possible that the effects of smells on mood and store evaluations escaped the customers' conscious awareness (e.g., Degel et al., 2001; Holland et al., 2005; de Groot et al., 2020a), making it more difficult to find mediation effects using these explicit measures. Future research could make use of VR techniques, controlled odor delivery, and implicit measures of mood and products aside from the current explicit measures. Also, the questionnaire was quite short to increase compliance, but this came at the expense of reliability. Some subscales consisted of only two items, whereas the appropriate scale length is three items or more. Another aspect concerns questions about mood (and arousal). It was not explicitly stated that the participants should report the level of happiness and tension at that very moment. Some people may have difficulties reporting their own mood (Lineweaver and Brolsma, 2014), causing them to answer the question based on their general mood or arousal from the past day, week or month. In future research the questionnaire should be pre-tested before using it as an instrument. Another notable facet is that on each of the subscales (mood, store evaluation, product evaluation, and staff evaluation), customers scored an average 6 on a 7-point scale, with staff getting an average rating of 6.6. These high scores may be a result of selection bias and social desirability. Regarding the former, only about one in six customers who bought an item at the second-hand clothing store completed the questionnaire. Of these individuals, all 57 reported the intention to come back to the store. It could be that only those individuals that were in a good mood, who had a positive view of the store, its products, and the staff, filled out this questionnaire. The high ratings could also stem from the customers showing social desirability, to please the staff (as the researchers were not present). This is possible since the questionnaires were filled out at the counter in close proximity to the staff. It was deliberately chosen to instruct the staff to point the customer's attention to the questionnaire, because the presence of researchers in the store can have a profound effect on the customers and the store personnel (Wood et al., 2006). Creating as much a realistic and natural setting as possible was precisely the aim of this research. Also, the researchers were aware of the hypotheses and could have modified their behavior to obtain the desired results if they would have been present. The store personnel, however, was indifferent to the hypotheses and the study's outcome and would therefore exert a negligible influence on the results. Hence, the current study was truly double blind.

A third limitation is the study design. Compared to a lab experiment, experimental control is obviously more difficult in a field experiment. It cannot be guaranteed that all individuals have been exposed to the same quantity of odor, for the same duration. Aside from that, customers' smell thresholds were unlikely to be identical, which may have caused that the odor was perceived above threshold by some and below their threshold of conscious reporting by others (Smeets and Dijksterhuis, 2014). Another factor is that smells were presented sequentially. To avoid contamination of the store odor by the diffused smells, the “no odor” condition was first in line, followed by the fresh linen scent and then—after a gap week—the vanilla sandalwood condition. A counterbalanced design would have been more optimal, but not crucial, as spending behavior was unrelated to extraneous factors like significantly better weather conditions in the vanilla sandalwood condition. Given the lower amount of money spent in the vanilla sandalwood condition (vs. fresh linen), it is also unlikely that customers were exposed to a residue of fresh linen smell, as this should have boosted spending. To conclude, higher external validity was traded off for potentially lower internal validity, or at least less experimental control. Yet, building on the numerous lab studies that have highlighted excellent human smell skills under sterile conditions (reviewed in e.g., McGann, 2017; de Groot et al., 2020b), examining the effects of odors in natural settings seems inevitable to discover how important smells are to our everyday lives. Admittedly, even though the effect of fresh linen scent on spending behavior was strong, the sample was small. Of the 300+ customers in the store, <20% completed the questionnaire, perhaps because there was no incentive and participation was truly voluntary. These issues may be overcome in future research by testing large samples from diverse backgrounds at different locations with incentives and using machine learning approaches to make sense of the rich, complex data, after which effective practical applications tailored to a specific store or situation could be developed.

### Implications

Different odors can affect the consumer, and even “nudge” them, in different ways according to the context, which has implications for successful application and ethics. Whereas a context-irrelevant pleasant odor can lift the customer's mood, a context-relevant pleasant odor has the additional benefit of increasing consumer spending by increasing the overall evaluation of the store. As such, the insights from this research can inform marked changes in the application of odors in consumer environments. This also warrants considerations about ethics, because once diffused in the store, smells cannot be avoided (Bradford and Desrochers, 2009; Emsenhuber, 2009). Whereas increasing a person's mood through smell may be a minor ethical issue, it becomes more severe if a smell can actually boost sales, and particularly if this concerns products that are not in line with a person's values (e.g., a non-sustainable purchase). Typically, nudges are subtle rearrangements of the choice architecture (Marchiori et al., 2017), and this could mean customers could be manipulated in a direction they are unaware of. To be ethically acceptable, House of Lords Science and Technology Select Committee (2011) for example have mentioned that people should be told about an intervention directly, which could hamper its effect through strategic control mechanisms on the part of the customer (especially with smells), or an intervention should be just noticeable by a perceptive person (Thaler and Sunstein, 2008; Marchiori et al., 2017). As odor thresholds vary markedly from person to person (e.g., Oleszkiewicz et al., 2019), the concentration of the diffused odor could be set at the average threshold level for a certain population. Furthermore, research has shown that putting a sign on the counter stating “we are helping you to make healthy choices” did not impact a healthy food choice nudge effectiveness (Kroese et al., 2015); therefore, a similar sign replacing “healthy” with “sustainable” could make customers aware of a possible smell intervention, which may (or may not) be in line with their core values (for them to judge). In sum, the greater knowledge on the workings of smells in built environments needs to be coupled to thinking critically about the ethical considerations that are surrounding their application, and these concerns may be different for different scents in different contexts.

## Conclusion

The current findings are promising for scent marketing in sustainable environments like a second-hand clothing store. A specific ambient scent almost doubled the sales by associating good qualities (“clean,” “hygienic”) with the products in the store. Contrary to much research, it was shown that consumer behavior is not simply impacted by diffusing a pleasant scent in the air; the smell needs to have a particular meaning with respect to the store or product context in which it is diffused. Hence, by keeping the rest of the store environment exactly the same and simply altering the regular store odor to fresh linen scent, customers could be nudged to buy more in this sustainable environment. Smells were initially neglected as a medium that would successfully contribute to preventing irreversible damage to our environment due to disposal issues; yet, here they were demonstrated effective, although more research is needed to chart smells' effectiveness in case of a *direct* comparison between sustainable vs. unsustainable choices. As the fashion industry is one of the most polluting industries in the world (Howell, 2021), the application of scents to facilitate sustainable behavior could eventually help at least a bit to put Earth Overshoot Day higher up the calendar.

## Data Availability Statement

The raw data supporting the conclusions of this article are permanently available on the Open Science Framework: https://osf.io/ax7yp/.

## Ethics Statement

Ethical review and approval was not required for the study on human participants in accordance with the local legislation and institutional requirements. The patients/participants provided their written informed consent to participate in this study.

## Author Contributions

The author confirms being the sole contributor of this work and has approved it for publication.

## Conflict of Interest

The author declares that the research was conducted in the absence of any commercial or financial relationships that could be construed as a potential conflict of interest.

## Publisher's Note

All claims expressed in this article are solely those of the authors and do not necessarily represent those of their affiliated organizations, or those of the publisher, the editors and the reviewers. Any product that may be evaluated in this article, or claim that may be made by its manufacturer, is not guaranteed or endorsed by the publisher.
