# Supplementary material for: Smells in Sustainable Environments: The Scented Silk Road to Spending
Source: Front Psychol. 2021 Aug 20;12:718279. doi: 10.3389/fpsyg.2021.718279 (PMC8417554; doi:10.3389/fpsyg.2021.718279)
Supplement: Supplementary file 1 [file Data_Sheet_1.pdf]

Supplementary materials

Appendix 1: Dutch Questionnaire with English translation of key terms and numbered items

Wat leuk dat u een aankoop heeft gedaan bij de *Green Label Store*! Momenteel wordt hier een onderzoek gedaan door studenten van de Universiteit Utrecht naar de winkelervaring in tweedehandswinkels. Het zou daarom erg fijn zijn als u deze vragenlijst in zou willen vullen. Mocht u deze al een keer eerder in hebben gevuld in deze winkel, geeft u dit dan alstublieft aan bij het personeel.
Wij willen graag benadrukken dat de data anoniem worden verwerkt. Uw gegevens worden vertrouwelijk behandeld en zijn nooit terug te leiden naar u. Vul de vragenlijst dus zo volledig en eerlijk mogelijk in – er zijn geen goede of foute antwoorden!
Wanneer u zich bedenkt nadat u een antwoord heeft ingevuld, wilt u dan het foute antwoord duidelijk doorstrepen en het goede antwoord alsnog invullen. Wilt u er ook goed op letten dat u bij iedere vraag een antwoord invult?

Hieronder volgen enkele stellingen. De antwoordmogelijkheden lopen stapsgewijs van links naar rechts. Er staat iedere keer aangegeven wat de linker- en rechterkant precies inhoudt. Omcirkel het cijfer dat voor u van toepassing is. Let erop dat u iedere keer maar één cijfer omcirkelt.

1. Wat is uw algemene indruk van deze winkel? [What is your general impression of this store?]

Niet leuk 1 2 3 4 5 6 7 Leuk
[Not nice] [Nice]

2. Hoe levendig vindt u deze winkel? [How stimulating do you find this store?]

Saai 1 2 3 4 5 6 7 Levendig
[Boring] [Stimulating]

3. Hoe interessant vindt u deze winkel? [How interesting do you find this store?]

Oninteressant 1 2 3 4 5 6 7 Interessant
[Uninteresting] [Interesting]

4. Hoeveel vertrouwen heeft u in de producten die in deze winkel worden verkocht? [How much trust do you have in the products being sold in the store?]

Weinig 1 2 3 4 5 6 7 Veel
[A little] [A lot]

5. Hoe voelt u zich op dit moment? [How do you feel at the moment?]

Ontspannen 1 2 3 4 5 6 7 Gespannen
[Relaxed] [Tense]

6. Wat vindt u van de kwaliteit van de producten in deze winkel? [What do you think of the quality of the products in the store?]

Lage kwaliteit 1 2 3 4 5 6 7 Hoge kwaliteit
[Low quality] [High quality]

7. Hoe voelt u zich op dit moment? [How do you feel at the moment?]

Ongelukkig 1 2 3 4 5 6 7 Gelukkig
[Unhappy] [Happy]

8. Hoe beoordeelt u het personeel van deze winkel? [How do you evaluate the personnel in this store?]

Onvriendelijk 1 2 3 4 5 6 7 Vriendelijk
[Unfriendly] [Friendly]

9. Heeft u enige twijfel over uw aanschaf gehad? [Did you have any doubts about your purchase?]

Geen twijfel 1 2 3 4 5 6 7 Twijfel

[No doubts] [Doubts]

10. Hoe komt de winkel op u over? [What's your impression of the store?]

Niet rommelig 1 2 3 4 5 6 7 Rommelig
[Not messy] [Messy]

11. Hoe hygiënisch vindt u de producten in deze winkel? [How hygienic are the products in the store?]

Onhygiënisch 1 2 3 4 5 6 7 Hygiënisch
[Not hygienic] [Hygienic]

12. Hoe heeft u het personeel ervaren? [How did you experience the personnel?]

Niet behulpzaam 1 2 3 4 5 6 7 Behulpzaam
[Not helpful] [Helpful]

13. Hoe voelt u zich op dit moment? [How do you feel at the moment?]

Geïrriteerd 1 2 3 4 5 6 7 Blij
[Annoyed] [Pleased]

14. Hoeveel geld heeft u vandaag besteed in deze winkel? [How much money have you spent in this store today?]

€ _____ , ____

15. Hoe vaak brengt u een bezoek aan tweedehands kledingwinkels? [How often do you visit a second hand clothing shop?]

- □ 1 keer per jaar (of minder) [Once a year (or less)]
- □ 2 keer per jaar [Twice a year]
- □ Een keer per kwartaal [Once per quarter]
- □ Maandelijks [Monthly]
- □ Wekelijks (of vaker) [Weekly (or more)]

16. Bent u, op basis van uw ervaring in deze winkel, van plan terug te komen naar deze of een soortgelijke kledingwinkel? [Are you, based on your experience in this store, planning to come back to this or a comparable store?]

- □ Ja [Yes]
- □ Nee [No]

17. U bent een… [U are a..]

- □ Vrouw [Woman]
- □ Man [Man]

18. Uw leeftijd is: ………………… [Your age is]

19. Heeft u op dit moment een verminderd reukvermogen door ziekte/verkoudheid etc. [Do you momentarily have a reduced sense of smell due to illness/cold etc.]

- □ Ja [Yes]
- □ Nee [No]

20. Heeft u bij binnenkomst een ander soort geur geroken dan u in deze winkel had verwacht? [Have you by entering this store smelled an odor that is different than what you had expected?]

- □ Ja [Yes]
- □ Nee [No]

Indien u ja heeft geantwoord op de vorige vraag, vult u dan alstublieft onderstaande vragen in. Wanneer u nee heeft geantwoord, kunt u deze laatste 5 vragen overslaan. [If you answered 'yes' tot he last questoin, please fill out the next questions. If you answered 'no', you can skip the final 5 questions.]

21. Wat voor een geur denkt u te hebben geroken? [What kind of smell did you perceive?]

………………………………….

22. Wat vindt u van de geur? [What do you think of this smell?]

Onplezierig [Unpleasant]	1	2	3	4	5	6	7	Plezierig [Pleasant]

23. Hoe toepasselijk vindt u deze geur voor de winkel? [How appropriate do you find the smell to be for this store?]

Niet toepasselijk [Not appropriate]	1	2	3	4	5	6	7	Toepasselijk [Appropriate]

24. Hoe intens heeft u deze geur ervaren? [How intense did you think the smell was?]

Niet intens [Not intense]	1	2	3	4	5	6	7	Intens [Intense]

25. Hoe bekend is deze geur voor u? [How familiar is this smell for you?]

Onbekend [Unfamiliar]	1	2	3	4	5	6	7	Bekend [Familiar]

Hartelijk bedankt voor het invullen van de vragenlijst!

Zoals hierboven te lezen is, worden uw gegevens vertrouwelijk behandeld. Bent u geïnteresseerd naar onze resultaten of wilt u meer informatie over dit onderzoek? Dan kunt u uw e-mailadres achterlaten op het formulier bij de kassa.

Appendix 2: Original Dutch questionnaire

Universiteit Utrecht

Wat leuk dat u een aankoop heeft gedaan bij de *Green Label Store*! Momenteel wordt hier een onderzoek gedaan door studenten van de Universiteit Utrecht naar de winkelervaring in tweedehandswinkels. Het zou daarom erg fijn zijn als u deze vragenlijst in zou willen vullen. Mocht u deze al een keer eerder in hebben gevuld in deze winkel, geeft u dit dan alstublieft aan bij het personeel. Wij willen graag benadrukken dat de data anoniem worden verwerkt. Uw gegevens worden vertrouwelijk behandeld en zijn nooit terug te leiden naar u. Vul de vragenlijst dus zo volledig en eerlijk mogelijk in – er zijn geen goede of foute antwoorden!
Wanneer u zich bedenkt nadat u een antwoord heeft ingevuld, wilt u dan het foute antwoord duidelijk doorstrepen en het goede antwoord alsnog invullen. Wilt u er ook goed op letten dat u bij iedere vraag een antwoord invult?

Hieronder volgen enkele stellingen. De antwoordmogelijkheden lopen stapsgewijs van links naar rechts. Er staat iedere keer aangegeven wat de linker- en rechterkant precies inhoudt. Omcirkel het cijfer dat voor u van toepassing is. Let erop dat u iedere keer maar één cijfer omcirkelt.

Wat is uw algemene indruk van deze winkel?
Niet leuk 1 2 3 4 5 6 7 Leuk

Hoe levendig vindt u deze winkel?
Saai 1 2 3 4 5 6 7 Levendig

Hoe interessant vindt u deze winkel?
Oninteressant 1 2 3 4 5 6 7 Interessant

Hoeveel vertrouwen heeft u in de producten die in deze winkel worden verkocht?
Weinig 1 2 3 4 5 6 7 Veel

Hoe voelt u zich op dit moment?
Ontspannen 1 2 3 4 5 6 7 Gespannen

Wat vindt u van de kwaliteit van de producten in deze winkel?
Lage kwaliteit 1 2 3 4 5 6 7 Hoge kwaliteit

Hoe voelt u zich op dit moment?
Ongelukkig 1 2 3 4 5 6 7 Gelukkig

Hoe beoordeelt u het personeel van deze winkel?
Onvriendelijk 1 2 3 4 5 6 7 Vriendelijk

Heeft u enige twijfel over uw aanschaf gehad?
Geen twijfel 1 2 3 4 5 6 7 Twijfel

Hoe komt de winkel op u over?
Niet rommelig 1 2 3 4 5 6 7 Rommelig

Hoe hygiënisch vindt u de producten in deze winkel?
Onhygiënisch 1 2 3 4 5 6 7 Hygiënisch

Hoe heeft u het personeel ervaren?

Niet behulpzaam	1	2	3	4	5	6	7	Behulpzaam

Hoe voelt u zich op dit moment?

Geïrriteerd	1	2	3	4	5	6	7	Blij

Hoeveel geld heeft u vandaag besteed in deze winkel?
€ _____ , ____

Hoe vaak brengt u een bezoek aan tweedehands kledingwinkels?
- □ 1 keer per jaar (of minder)
- □ 2 keer per jaar
- □ Een keer per kwartaal
- □ Maandelijks
- □ Wekelijks (of vaker)

Bent u, op basis van uw ervaring in deze winkel, van plan terug te komen naar deze of een soortgelijke kledingwinkel?
- □ Ja
- □ Nee

U bent een…
- □ Vrouw
- □ Man

Uw leeftijd is: ………………..

Heeft u op dit moment een verminderd reukvermogen door ziekte/verkoudheid etc.
- □ Ja
- □ Nee

Heeft u bij binnenkomst een ander soort geur geroken dan u in deze winkel had verwacht?
- □ Ja
- □ Nee

Indien u ja heeft geantwoord op de vorige vraag, vult u dan alstublieft onderstaande vragen in. Wanneer u nee heeft geantwoord, kunt u deze laatste 5 vragen overslaan.

Wat voor een geur denkt u te hebben geroken?
………………………………..

Wat vindt u van de geur?

Onplezierig	1	2	3	4	5	6	7	Plezierig

Hoe toepasselijk vindt u deze geur voor de winkel?

Niet toepasselijk	1	2	3	4	5	6	7	Toepasselijk

Hoe intens heeft u deze geur ervaren?

Niet intens	1	2	3	4	5	6	7	Intens

Hoe bekend is deze geur voor u?

Onbekend	1	2	3	4	5	6	7	Bekend

Hartelijk bedankt voor het invullen van de vragenlijst!

Zoals hierboven te lezen is, worden uw gegevens vertrouwelijk behandeld. Bent u geïnteresseerd naar onze resultaten of wilt u meer informatie over dit onderzoek? Dan kunt u uw e-mail adres achterlaten op het formulier bij de kassa.
